# The transcriptomic signature of fasting murine liver

**DOI:** 10.1186/1471-2164-9-528

**Published:** 2008-11-06

**Authors:** Milka Sokolović, Aleksandar Sokolović, Diederik Wehkamp, Emiel Ver Loren van Themaat, Dirk R de Waart, Lisa A Gilhuijs-Pederson, Yuri Nikolsky, Antoine HC van Kampen, Theodorus BM Hakvoort, Wouter H Lamers

**Affiliations:** 1 AMC Liver Center, Academic Medical Center, University of Amsterdam, The Netherlands; 2 Department of Medical Biochemistry, Academic Medical Center, University of Amsterdam, The Netherlands; 3 Bioinformatics Laboratory, Academic Medical Center, University of Amsterdam, The Netherlands; 4 GeneGo, Inc., St. Joseph, MI, USA

## Abstract

**Background:**

The contribution of individual organs to the whole-body adaptive response to fasting has not been established. Hence, gene-expression profiling, pathway, network and gene-set enrichment analysis and immunohistochemistry were carried out on mouse liver after 0, 12, 24 and 72 hours of fasting.

**Results:**

Liver wet weight had declined ~44, ~5, ~11 and ~10% per day after 12, 24, 48 and 72 hours of fasting, respectively. Liver structure and metabolic zonation were preserved. Supervised hierarchical clustering showed separation between the fed, 12–24 h-fasted and 72 h-fasted conditions. Expression profiling and pathway analysis revealed that genes involved in amino-acid, lipid, carbohydrate and energy metabolism responded most significantly to fasting, that the response peaked at 24 hours, and had largely abated by 72 hours. The strong induction of the urea cycle, in combination with increased expression of enzymes of the tricarboxylic-acid cycle and oxidative phosphorylation, indicated a strong stimulation of amino-acid oxidation peaking at 24 hours. At this time point, fatty-acid oxidation and ketone-body formation were also induced. The induction of genes involved in the unfolded-protein response underscored the cell stress due to enhanced energy metabolism. The continuous high expression of enzymes of the urea cycle, malate-aspartate shuttle, and the gluconeogenic enzyme Pepck and the re-appearance of glycogen in the pericentral hepatocytes indicate that amino-acid oxidation yields to glucose and glycogen synthesis during prolonged fasting.

**Conclusion:**

The changes in liver gene expression during fasting indicate that, in the mouse, energy production predominates during early fasting and that glucose production and glycogen synthesis become predominant during prolonged fasting.

## Background

Abstinence or absence of food requires the body to recruit metabolites from pre-existing stores. Based on the rate of weight loss, nitrogen excretion, concentration of plasma metabolites and resting metabolic rate, the body is thought to pass through three successive adaptive phases during fasting [1] that have been associated with the primary fuel that is putatively available to the tissues (e.g [2-5]). During the brief postabsorptive period, the rate of weight loss is relatively high (~24% per day in mice [6], ~10% per day in rats [7,8], and ~2% in humans [9]). The decreasing insulin levels induce glycogenolysis (primarily muscle and liver) and lipolysis [10,11] to support circulating glucose, triglyceride and cholesterol levels [8]. During the subsequent "coping" phase, the loss of body mass is slower (~7% per day in mice, ~6% per day in rats [7], and ~1% in humans [12]). This state, which can be maintained for several weeks in humans [13,14], for almost a week in rats [7], and for 2–3 days in mice [6], is thought to depend, at least in humans, on lipids as the main fuel source. However, amino-acid oxidation and, hence, protein catabolism remains necessary for continuous anaplerosis of the TCA cycle [14]. It is widely accepted that muscle is a main source of amino acids from protein catabolism, that protein catabolism is maintained by an increased in the circulating levels of glucocorticosteroids, and that glutamine and alanine are the main carriers of this energy source [15,16] to the intestine, liver and kidney [2,5,7,17-19]. As a result, total splanchnic glucose production amounts to approximately 80 grams daily in humans after several weeks of starvation [13]. Despite this enhanced glucose production, but reflecting the enhanced fatty-acid oxidation and ketone-body synthesis in muscle and splanchnic region [5,20], the brain gradually switches to ketone-body oxidation after several weeks of starvation [21,13]. During the preterminal phase, finally, the rate of loss of body weight may increase again (~9% in rats [7]). Because the fat stores are depleted, proteolysis remains the sole, nonsustainable source of fuel.

The maintenance of the fuel supply during fasting requires an extensive exchange of metabolites from organs that break down the stores of fats or proteins to organs that consume these metabolites. This exchange mainly occurs as glucose, lactate, amino acids, triglycerides and ketone bodies. The question that arises from these global findings concerns the contribution of individual organs to the whole-body adaptive response to fasting. Our previous study of the effects of fasting on the small intestine [6] suggested, in comparison with that of liver [22] and muscle [23,24], an organ-specific response to fasting. Our study [6] included both shorter and longer periods of fasting than earlier published studies [22-24]. The aim of the current study was, therefore, to determine the characteristics of gene-expression profile of mouse liver between 0 and 72 hours of fasting, using a genome-wide transcriptomics approach. Our findings show that the adaptive response of the liver peaks around 24 hours after food withdrawal and, unexpectedly, declines thereafter. The major components of the response were fatty-acid β-oxidation and ketone-body synthesis, and oxidative and energy metabolism during the first 24 hours of fasting, and glycogen synthesis and the urea cycle throughout the entire fasting period.

## Results

### Effects of food withdrawal on liver structure

**Figure 1:**
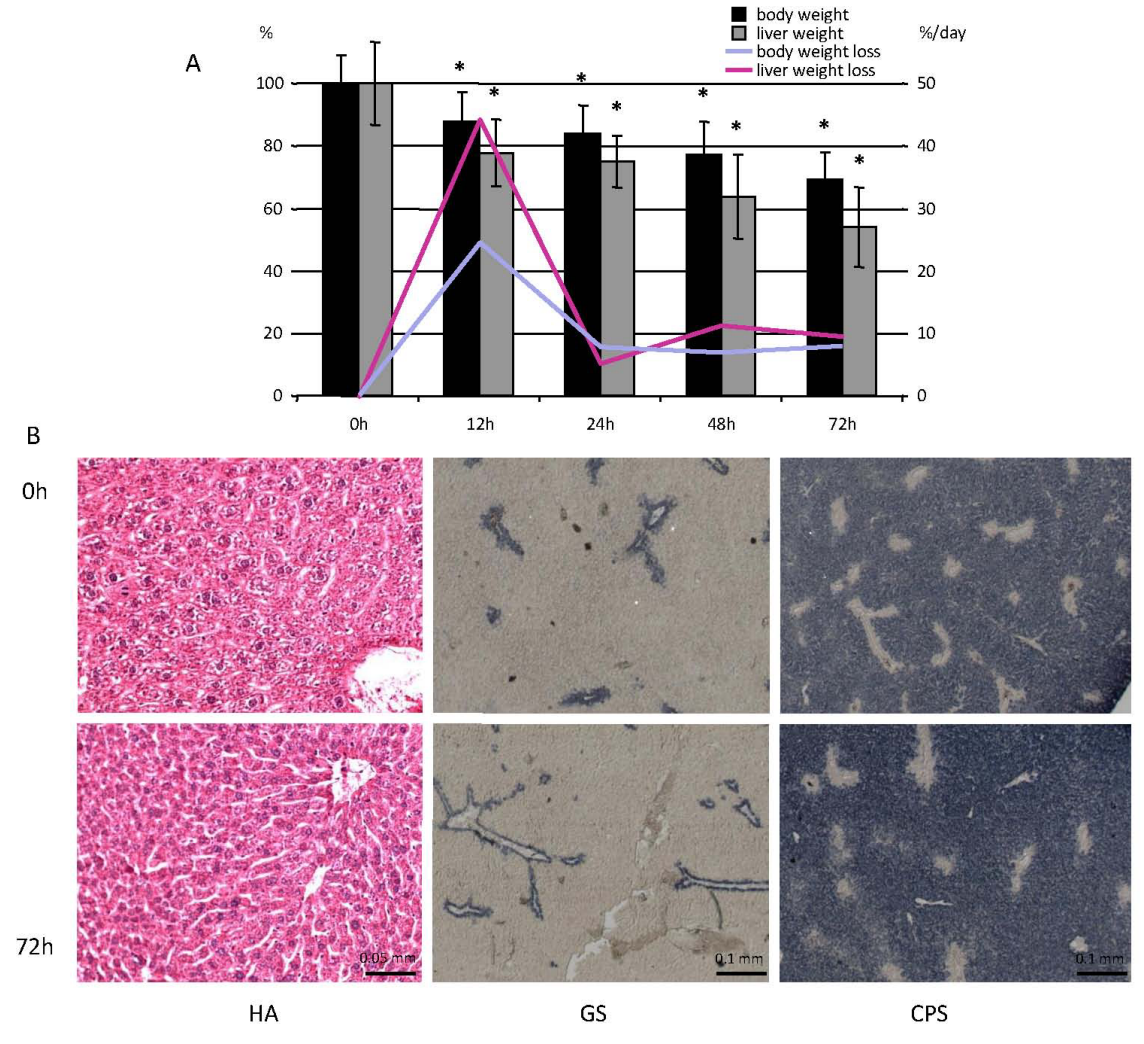
**Macro- and microscopic analysis of the fasting liver**. A) Change in whole-body and liver weight during fasting as percentage of fed weight (n ≥ 8). Asterisks label significant changes (P < 0.01). The blue and pink lines represent the daily percentual change in body and liver weight, respectively, with the percent weight loss per day shown on the secondary y-axis. B) Histology of fed and 72 hour-starved livers (upper and lower panel, respectively). The sections were stained with hematoxylin and azophloxin (HA), and for the presence of glutamine synthetase (GS; pericentral expression) and carbamoylphosphate synthetase (CPS; periportal expression). The figures show that lobular architecture and metabolic zonation are unaffected by fasting. Bars: 0.1 mm.

During the first 12 hours of fasting, mice lost ~12% of their body weight (that is, 24% if expressed on a per-day basis). Thereafter, weight loss remained steady at a rate of ~7% per day, so that mice had lost ~30% of their initial weight after 72 hours of fasting (Figure 1A). Note that we expressed daily differences in the rate of weight loss on a per-day basis to define a common denominator for the 12 h- and 24 h-fasted animals. Liver wet weight declined more than body weight (Figure 1A), especially during the first 12 hours of fasting, and amounted to ~44, ~5, ~11 and ~10% per day after 12, 24, 48 and 72 hours of fasting, respectively. After 72 hours, the liver had, therefore, lost almost 50% of its initial weight. The basic architecture of the liver lobules (Figure 1B, HA) and the zonation of gene expression as studied by the expression of glutamine synthetase and carbamoylphosphate synthetase (Figure 1B, GS and CPS) remained unaffected. Staining for the appearance of active-caspase 3 revealed no changes in the number of apoptotic cells upon fasting, not even after 72 hours (data not shown). In agreement, the apoptotic genes that were represented on the microarrays showed no significant change in expression in fasted compared to fed mice. Since there was no reduction in the number of liver cells during fasting, we took two approaches to estimate the decrease in average cell size. The summation of 50 hepatocyte diameters in three 72 hours fasted and three control animals, amounted to 25% reduction in cell diameter. Based on the liver wet weight, the average cell diameter decreased 20% in the fasted animals.

### Effects of fasting on metabolism

**Figure 2:**
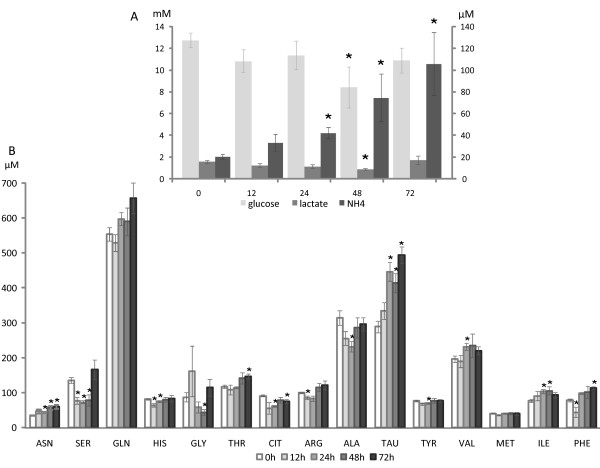
**Changes in plasma metabolite concentrations during fasting**. A) Glucose and lactate (mM, primary Y-axis), and ammonia concentrations (μM, secondary Y-axis) after 0, 12, 24, 48 and 72 hours of fasting. B) Adaptive changes in concentrations of a selection of amino acids during fasting (those without significant change between any two time points were left out). For all the metabolites measured: 8 < n < 12; bars represent SEM and the asterisks identify significant changes (P < 0.05) in comparison to the fed condition.

Ammonia levels had increased 2.0-, 3.7- and 5.2-fold after 24, 48 and 72 hours of fasting, respectively (P < 0.005; Figure 2A). Glucose and lactate concentrations remained stable until 48 h of fasting, but decreased 34 and 43%, respectively (P < 0.05 and 0.005, respectively, Figure 2A) in the next 24 hours. The plasma concentration of many amino acids changed at some time point of fasting, but only the changes in the concentration of taurine showed a trend with time (Figure 2B and Supplementary Table 1, Additional file 1). Accumulation of taurine helps protect cells from hypertonicity [25], as may occur during shrinkage of fasting hepatocytes.

**Table 1:** Top 10 canonical pathways influenced by fasting

**pathway**	**pathway group**	**p- value**	**genes**
**glycolysis and gluconeogenesis**	metabolic maps/carbohydrate metabolism	2.32e-07	9/36
**urea cycle**	metabolic maps/amino-acid metabolism	2.61e-07	8/27
**PPARα regulation of lipid metabolism**	regulation of metabolism/regulation of lipid metabolism	3.57e-07	8/28
**peroxisomal straight-chain fatty-acid β-oxidation**	metabolic maps/lipid metabolism	2.51e-06	5/10
**mitochondrial long-chain fatty- acid β-oxidation**	metabolic maps/lipid metabolism	2.74e-06	6/17
**metabolism of sulphur-containing amino acids**	metabolic maps/amino-acid metabolism	4.02e-06	6/18
**peroxisomal branched-chain fatty- acid oxidation**	metabolic maps/lipid metabolism	8.04e-06	6/20
**taurine metabolism**	metabolic maps/amino-acid metabolism	1.96e-05	6/23
**mitochondrial unsaturated fatty- acid β-oxidation**	metabolic maps/lipid metabolism	2.69e-05	5/15
**TCA**	metabolic maps/amino-acid metabolism	1.25e-04	4/20

Canonical pathways represent a set of about 500 signalling and metabolic maps in the MetaCore suit. All maps are drawn from scratch by GeneGo annotators and manually curated.

### Global gene-expression profile in the liver

**Figure 3:**
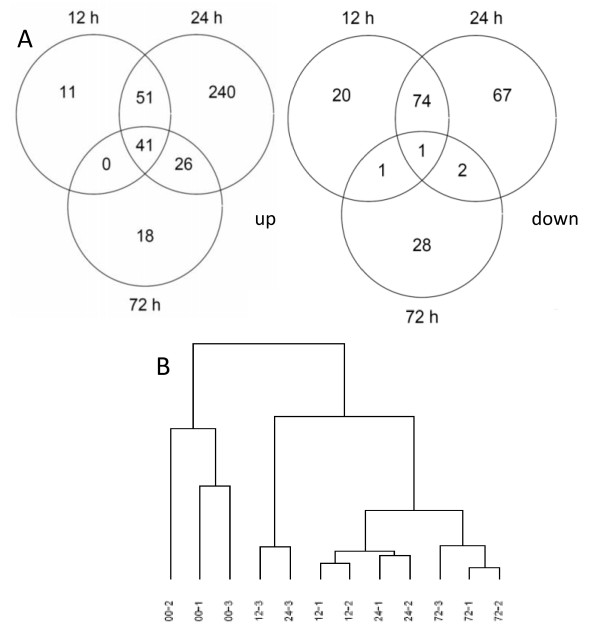
**Number of genes in the liver that are affected by fasting**. A) Number of differentially expressed genes (≥ 1.4-fold change in expression; P < 0.01) at each time point studied. The left-sided Venn diagram shows the number of up-regulated and the right-sided diagram the number of down-regulated genes. Genes that were changed in expression at more than one time point are shown in the overlapping areas. B) Supervised hierarchical cluster based on correlation and complete linkage.

To gain a comprehensive overview of the physiological response of the liver to fasting, whole-genome measurements were made. Compared to the fed group, 201, 504 and 119 transcripts, including expressed sequence tags and RIKEN sequences, met our boundary condition for significance (≥ 1.4-fold change with P < 0.01) after 12, 24, and 72 hours of fasting, respectively (Figure 3A; for a complete list of more than 1.4-fold up- or downregulated genes, see Additional file 2). The dendrogram generated by supervised hierarchical clustering (Figure 3B) shows a clear separation between fed and fasted conditions. Among the arrays coming from fasted animals, those from 72 hours stand out, while the branches of the two earlier time points are intertwined, indicating that expression profiles are rather similar after 12 and 24 hours of fasting. This is also reflected in the Venn diagrams where the overlap between 12 and 24 hours is larger than the overlap with 72 hours.

### Global analysis reveals a strong early and an abated late response to fasting

**Figure 4:**
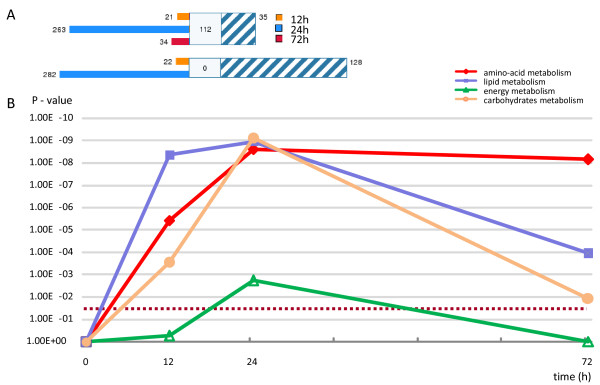
**Adaptive changes in metabolic processes in the liver during fasting as analyzed by MetaCore™ software**. A) The gene content imported to MetaCore™ is aligned between the time points. The parameters for comparison are ≥ 1.4 fold change and P < 0.01, and the annotation allowed for 56% of such genes to be linked. The *unique *genes changed at each of the time points are marked as colored bars (orange, blue and red for 12, 24 and 72 hours, respectively). The set of *common *genes, changed in all three conditions, is shown in blue-white hatching. The middle white box represents the *similar *genes (present in 2 out of 3 data points). The upper panel represents differentially regulated genes in all three time points of fasting, while the lower one shows uniquely and commonly differentially expressed genes for 12 and 24 hours of fasting. B) Four groups of metabolic processes changed significantly in response to fasting, with the response of all peaking at 24 hours. The Y-axis shows the significance of change, while the X-axis represents duration of fasting. The P-values of the pathways are calculated using the hypergeometric distribution, where the P-value represents the probability of a particular mapping arising by chance, given the numbers of genes in the set of all genes in pathways, genes in a particular pathway, and genes in the present experiment. The pathways are grouped into processes as defined in MetaCore™ (version 4.3, build 9787). The dotted line represents the 0.05 significance threshold.

We used GenMAPP and, in particular, MetaCore™ software to deduce the biological processes that change with an increasing duration of fasting from the liver transcriptome data. In MetaCore, the degree of association of the uploaded datasets with predefined metabolic pathways is defined by P-values, with lower P-values being more relevant. The expression of 465 genes that met our thresholds (56%) could be linked to the MetaCore™ suite. Their distribution across time points is shown in Figure 4A. The graphs show the numbers of unique, similar and common genes for all three, and for two initial time points separately, showing that the response to fasting at 24 hours was similar to, but more pronounced than that at 12 hours.

We performed gene-set enrichment analysis in three different functional ontologies using MetaCore™: cellular processes, biological processes and canonical pathways. Based on the Gene Ontology categorization of cellular processes, fasting predominantly affected the metabolic processes, in particular the carboxylic-acid metabolizing processes, lipid and glucose metabolism. The enrichment analysis for biological processes showed, more specifically, that genes involved in amino-acid, lipid, carbohydrate and energy metabolism responded most significantly to fasting (Figure 4B). The graph presents P-values as parameter of the likelihood that coordinate changes in the pathways shown were indeed present at the different time points of fasting. As statistical parameter, the P-value encompasses no variation. The changes in all processes except amino-acid metabolism showed a response that peaked at 24 hours after food withdrawal and declined thereafter. The response during the late phase of fasting was dominated by amino-acid metabolism, although lipid and carbohydrate metabolism remained significantly regulated. The Figure further reveals that the changes in energy metabolism were significant at 24 hours of fasting only. The common denominator of the overall fasting response was, therefore, metabolism of amino acids, carbohydrates, and lipids.

### Regulated pathways

Since the global analysis does not reveal a direction in the changes and lacks functional detail, we scrutinized the pathways with most pronounced regulation for functional implications. A list of the 10 top-scoring canonical pathways, shown in Table 1, points to gluconeogenesis, urea synthesis, and PPARα-regulated fatty-acid oxidation as the major characteristics in the response of the liver to fasting.

### Amino-acid catabolism and urea synthesis

**Figure 5:**
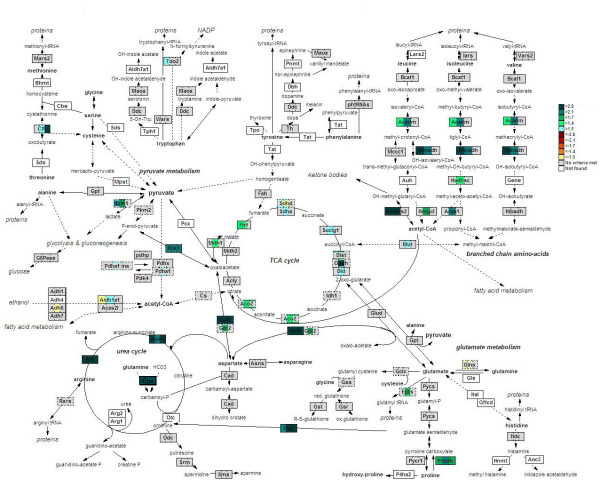
**Amino-acid catabolism in fasting liver**. The map was created in the GenMAPP suite to show a comprehensive overview of amino-acid metabolism in response to fasting. Warm colors (from yellow to red) represent down-regulation, while cold colors (light blue to dark green) indicate an induction (see scale on the right border of the figure). Gray indicates no significant change. Genes not coupled to reporters on the array are shown in white. Genes represented by more than one sequence on the array are shown in dash-lined boxes, with the level of change depicted by the colored line surrounding the field. Each gene-box is split into 3 units, representing (from left to right) a change in expression after 12, 24 and 72 hours of fasting compared to fed animals.

Of all the pathways studied in the liver, the adaptive changes in amino-acid metabolism persisted throughout the fasting period (Figure 4B). Of the enzymes in this group, those of the urea cycle were upregulated at all three time points (Figure 5). Among the genes consistently affected were argininosuccinate synthetase 1 (*Ass1*, *Assy*; 3.7-, 2.5- and 4.5-fold upregulated) and argininosuccinate lyase (*Asl*, *Arly*; 5.0-, 5.8-, and 12-fold upregulated at 12, 24 and 72 hours, respectively. The first and rate-determining enzyme of urea cycle, carbamoylphosphate synthetase (*Cps*), was not represented on the microarrays, but its expression level, as estimated by qPCR, was increased 3.5-fold at all 3 time points (manually added to Figure 5). Urea synthesis occurs in periportal hepatocytes, whereas ammonia detoxification via glutamine synthesis occurs pericentrally. Genes for the pericentral enzymes ornithine-aminotransferase (*Oat*) and proline dehydrogenase (*Prodh*), which provide glutamate for glutamine synthesis, were upregulated 2.5-, 3.0- and 3.0-fold and 2.1-, 2.5- and 2.0- fold at 12, 24 and 72 hours, respectively. Glutamine synthetase (Glns) itself was, however, not regulated.

Remarkably, the expression of amino-acid catabolizing enzymes themselves was barely affected by fasting. Only the degradation of branched-chain keto-acids (products of branched-chain amino-acid transamination elsewhere) was upregulated, as shown by the upregulation of acetyl-coenzyme A dehydrogenase, medium chain *(Acaddm)*, enoyl-coenzyme A, hydratase/3-hydroxyacyl-coenzyme *(Ehhadh)*, hydroxyacyl-coenzyme A dehydrogenase, short chain *(Hadhsc)*, acetyl-coenzyme A acyltransferase 1 *(Acaa1)*, and 3-hydroxy-3-methylglutaryl-coenzyme A lyase *(Hmgcl) *– all within first the 24 hours (Figure 5). This finding suggests that the adaptations in amino-acid catabolism during fasting mainly occur outside the liver. Since neither glutamate-pyruvate transaminase nor ammonia-inducible liver glutaminase were upregulated, the capacity of the liver to deaminate the amino-carriers alanine and glutamine must have been sufficient.

### TCA cycle and electron-transport chain

**Figure 6:**
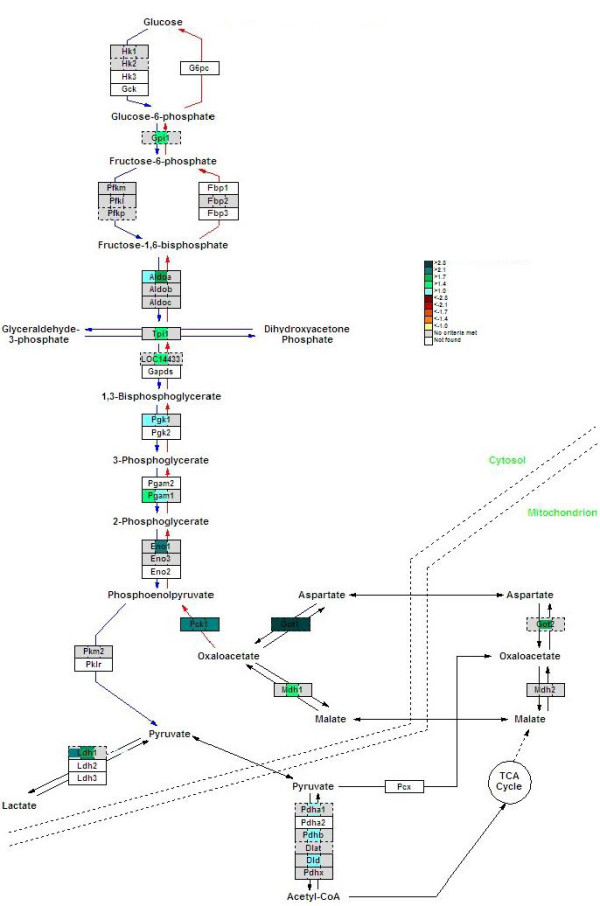
**Fasting upregulates genes of the malate-aspartate shuttle and the gluconeogenic enzyme *Pepck1 *in mouse liver**. The color code of the GenMAPP view is the same as in Figure 5.

The strong induction of the urea cycle suggests a strong stimulation of amino-acid oxidation or gluconeogenesis. In agreement with this hypothesis, both the expression of enzymes of the tricarboxylic-acid (TCA) cycle and oxidative phosphorylation were induced in fasted liver, again mainly at 24 hours. Aconitase 2 (*Aco2*), isocitrate dehydrogenase 3β (NAD^+^) (*Idh3b*), oxoglutarate dehydrogenase (*Ogdh*), dihydrolipoamide S-succinyltransferase (*Dlst*), fumarate hydratase 1 (*Fh1*) and malate dehydrogenase 1 (*Mdh1*) were all upregulated at 24 hours of fasting (1.9-, 1.5-, 3.1-, 2.0-, 1.4- and 1.6-fold, respectively; Figures 5 and 6), indicating an increased capacity of the cycle. *Dlst *and *Fh1 *were 2.0 and 1.6 times induced at 12 hours of fasting, while *Aco2 *expression was also 1.8-fold increased at 72 hours.

**Figure 7:**
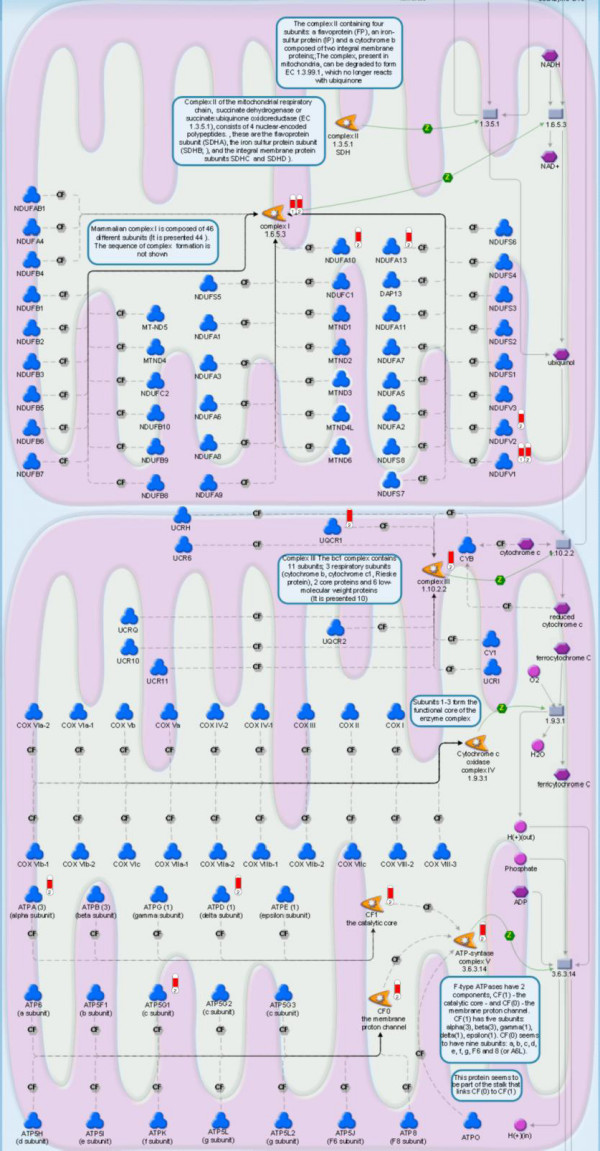
**Electron-transport chain**. Experimental data are visualized on a MetaCore map as blue (for downregulation) and red (upregulation) histograms ('thermometers'). The height of the histogram corresponds to the relative expression value for a particular gene, with numbers 1, 2 and 3 representing 12, 24 and 72 hours of fasting, respectively. A legend for the MetaCore™ canonical pathways is provided in Additional file 3.

**Figure 8:**
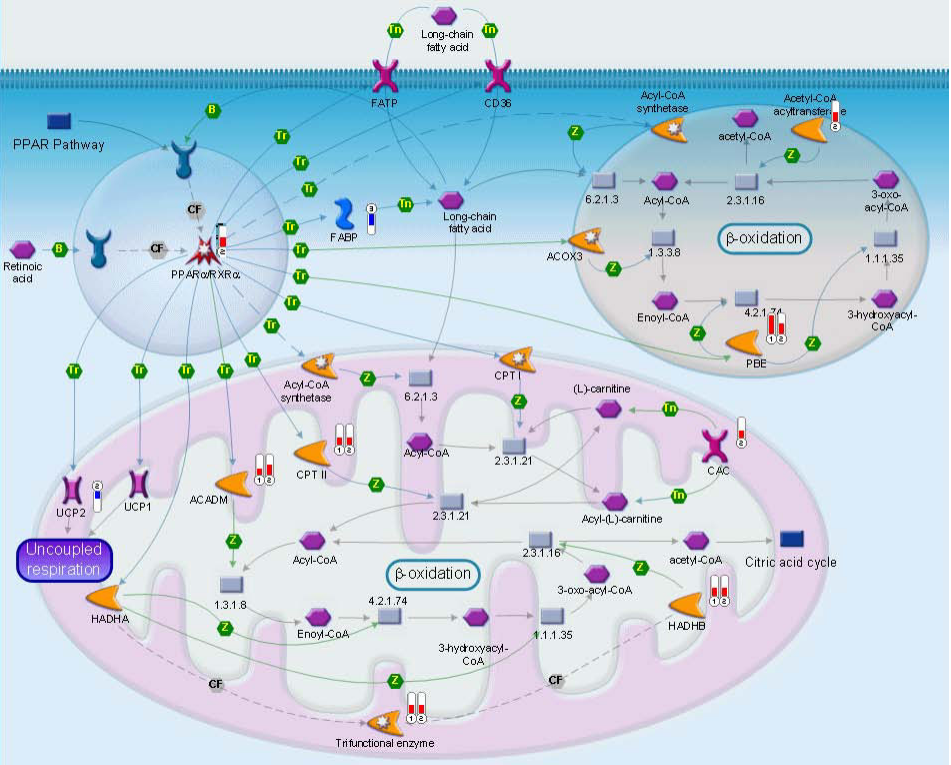
**PPARα regulation of lipid metabolism in fasting**. The figure description is the same as in Figure 7.

In agreement with an increased capacity of the TCA cycle, the expression of the genes of the electron-transport chain was strongly stimulated (Figure 7; a legend for the MetaCore canonical pathways is provided in Additional file 3). Four genes belonging to NADH-ubiquinone oxidoreductase complex: NADH dehydrogenase [ubiquinone] 1α subcomplex subunit 10 (*Ndufa10*), NADH dehydrogenase [ubiquinone] 1α subcomplex subunit 13 (*Ndufa13*), NADH dehydrogenase [ubiquinone] flavoprotein 1 (*Ndufv1*) and NADH dehydrogenase [ubiquinone] flavoprotein 2 (*Ndufv2*), were all approximately 1.6-fold upregulated. Expression of the genes of the ATP synthase complex, ATP synthase subunit α (*Atp5a1*), ATP synthase δ chain (*Atp5d*) and ATP synthase lipid-binding protein (*Atp5g1*), was 1.6–1.9-fold induced at 24 hours. Ubiquinol-cytochrome-c reductase complex core protein 1 (*Uqcrc1*) was 2.2-fold upregulated after 24 hours, whereas the energy-dissipating uncoupling protein 2 (*Ucp2*) was 1.8-fold downregulated at this time point (Figure 8). Taken together, these data indicate that the capacity for ATP synthesis in the liver is strongly upregulated during the first day of food deprivation.

### Gluconeogenesis

Phosphoenolpyruvate carboxykinase 1 (*Pepck1*), a key enzyme in the gluconeogenic route, was upregulated 2.0-, 2.5- and 2.7-fold on the microarrays and 3.2-, 3.2, and 2.9-fold in the qPCR measurements at 12, 24 and 72 hours of fasting, respectively (Figures 5, and 6, and Table 2). Cytosolic glutamate oxaloacetate transaminase 1 (*Got1*) was also strongly upregulated at all three time points (5-, 6-, and 21-fold). In addition, malate dehydrogenase (*Mdh*) and mitochondrial glutamate oxaloacetate transaminase (*Got2*) were induced (1.6- and 1.8-fold respectively). Together, these data suggest an increased capacity of the malate-aspartate shuttle across the mitochondrial membrane, which would accommodate an enhanced carbon flux from the mitochondria.

**Table 2:** Comparison of intestinal and liver *Pepck1 *expression in fasting by qRTPCR, expressed in relative units after normalization by 18 S expression (n = 6)

**time (h)**	**small intestine**	**liver**	**ratio**
**0**	18	85	0.21
**12**	15	278	0.05
**24**	51	277	0.18
**72**	128	247	0.52

All other steps that were affected by fasting were shared by the glycolytic and gluconeogenic pathways and were regulated during the first day of fasting only (Figure 6). Phosphoglycerate mutase 1 (*Pgam1*) was 1.4-fold upregulated at 12 hours of fasting, while glucosephosphate isomerase 1 (*Gpi1*), aldolase 1A isoform (*Aldoa*), triosephosphate isomerase 1 (*Tpi1*), glyceraldehyde-3-phosphate dehydrogenase (*Gapdh*) and enolase 1α (*Eno1*) were 1.5-, 1.7-, 1.5-, 1.7-, 2.1- fold upregulated at 24 hours of fasting, respectively. These data indicate that the enhanced capacity of the gluconeogenic pathway would largely depend on enhanced TCA and malate-aspartate cycling, and that this adaptive response in gene expression might be restricted to a single day in the mouse.

### Liver glycogen accumulation upon prolonged fasting

**Figure 9:**
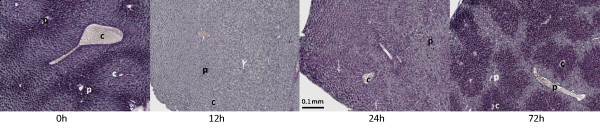
**Glycogen storage in mouse liver during fasting**. In fed liver, periportal hepatocytes contain most glycogen (left panel). Twelve hours of fasting totally depletes the glycogen stores, but at 24 hours, glycogen starts to re-accumulate and has accumulated to high levels in pericentral hepatocytes after 72 hours of fasting (right panel). Portal and central veins are depicted by letters "p" and "c", respectively. Bar: 0.1 mm.

The near total return to "normalcy" of gene expression at 72 hours (only the genes for urea cycle enzymes, glutamate-synthesizing enzymes, and Pepck1 remained induced) was striking. Because glucose-6-phosphatase expression was not upregulated, we explored the possibility that glucose precursors were channelled into glycogen. As expected, (amylase-sensitive) periodic acid-Schiff (PAS) staining showed the complete disappearance of glycogen from the liver after 12 hours of fasting (Figure 9), but some staining had returned at 24 hours and intense staining was seen in 72-hours fasted liver. Whereas glycogen was localized around the portal veins in fed liver, it was deposited exclusively around the central veins after 72 hours of fasting, with sharp borders towards the empty cells.

### Fatty-acid catabolism and ketone-body synthesis

The enhanced expression of fatty-acid catabolizing enzymes was also limited to the initial phase of fasting. The expression of the transcription factor *Pparα*, a major regulator of fatty-acid oxidation, was 2.1-fold upregulated at 24 hours of fasting (Figure 8). Furthermore, the mitochondrial carnitine/acylcarnitine fatty-acid translocase (*Cac *or *Slc25a20*) was 1.6-fold upregulated at 12 hours, while carnitine palmitoyltransferase 2 (*Cpt2*) on the inner mitochondrial membrane was 1.8-fold upregulated at both 12 and 24 hours of fasting. However, the expression of *Cpt1*, which is present on the outer mitochondrial membrane and is sensitive to malonyl-CoA inhibition, remained unchanged. The 4 acyl-coenzyme A dehydrogenases (*Acad -v, -l, -m and -sh*), involved in oxidation of very long-, long-, medium- and short-chain fatty acids, were all upregulated in the first 24 hours of fasting (1.5–2.6 fold). The β-subunit of the trifunctional protein (*Hadhb*) was 2- and 2.1-fold upregulated at 12 and 24 hours, respectively, while another subunit, hydroxyacyl-coenzyme A dehydrogenase, short chain (*Hadhsc*) showed increased expression after 24 hours of fasting only, indicating altogether a strong stimulation of fatty-acid oxidation at the gene-expression level during the first day of fasting.

The expression of HMGCoA synthase 2 (*Hmgcs2*) was also strongly stimulated during the first day of fasting (3.4- and 2.9-fold at 12 and 24 hours, respectively; Figure 5), indicating an increased capacity of the synthesis of ketone bodies from acetyl-CoA. This process is further facilitated by increased expression of genes involved in branched-chain keto-acid degradation (*Acadm, Hadhsc *and *Ehhadh*; see section on amino-acid catabolism) at 12 and 24 hours. Interestingly, neither the cytoplasmic HMGCoA synthase (*Hmgcs1*) nor HMGCoA reductase (*Hmgcr*), the key enzyme in de novo cholesterol synthesis pathway [26], have changed the expression levels in fasted liver.

Among the genes involved in fatty-acid synthesis, enoyl coenzyme A hydratase domain containing 3 (*Echdc3*) was 1.6 and 1.7-fold downregulated at 12 and 24 hours, while stearoyl-coenzyme A desaturase 1 (*Scd1*) showed a 2.6-fold decrease in expression at 72 hours of fasting. These data underscore the importance of enhanced lipid catabolism in the liver, which, in the mouse, apparently occurs during the first day of fasting only.

### Oxidative stress and unfolded protein response

The enhanced expression of TCA cycle and oxidative-phosphorylation enzymes often causes oxidative stress. Indeed, cytosolic superoxide dismutase (*Sod1*) was 2.2-fold upregulated after 24 hours, and the early growth response protein 1 (*Egr1*), its transcriptional regulator [27], 2.9-fold. Furthermore, catalase (*Cat*) and stress-regulated mitogen-activated protein kinase 14 (*Mapk14*) were both 1.4-fold upregulated at this time point. In addition, metallothionein 1 gene, known to be involved in protection against oxidative stress and metal toxicity [28], was intensely upregulated (8.6-, 5.5- and 13.5-fold, at 12, 24 and 72 hours, respectively).

**Figure 10:**
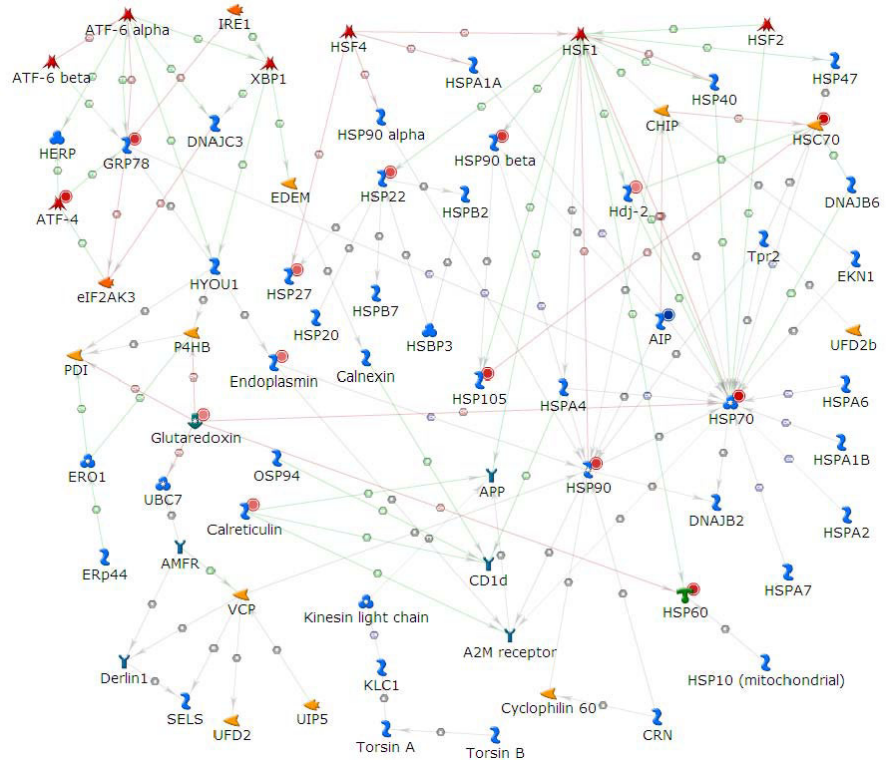
**The unfolded-protein response in fasting**. The network was generated and linked with available experimental data in the MetaCore™ suite. Nodes with red or blue circles in top right corner of the network objects, represent up- or down-regulation, respectively, with the shade indicating the intensity of the change. Detailed legend for MC networks is provided in Additional file 3.

**Figure 11:**
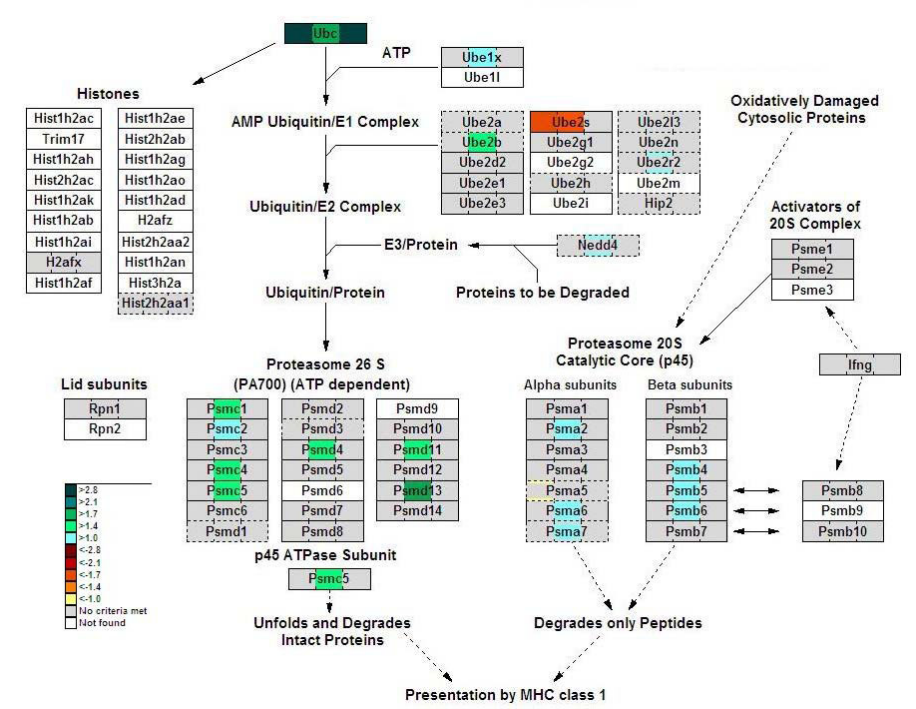
**Proteasome degradation in fasting**. The colour code of the GenMAPP view is the same as in Figure 5.

Interestingly, the 3 top-scoring processes obtained from a biological-process enrichment analysis all belonged to the unfolded-protein response (endoplasmic reticulum (ER) stress). To present the relevant data in a single figure, we created a network using the shortest-path algorithm (Figure 10). The resulting network provides links based on the known interaction data between the nodes from the query data set, and also between the nodes that regulate the given genes or metabolites. It shows 8 heat-shock and 6 other proteins, all upregulated 1.5–2.5 fold, indicating upregulation of this stress-response pathway in fasted liver. Downstream of the ER stress pathway, proteasome degradation was also upregulated, but again only after 24 hours of fasting (Figure 11). A list of these and some additional genes regulated in the ER stress and proteasome degradation, with their change level, is shown in Table 3.

**Table 3:** Genes involved in the protein-folding response and oxidative stress that are regulated by fasting

**gene name**	**gene symbol**	**12 h**	**24 h**	**72 h**
**AH receptor-interacting protein**	*Aip*	-1.78	-1.63	
**Cyclic AMP-dependent transcription factor ATF-4**	*Atf4*			2.60
**Calreticulin precursor**	*CalR*		1.67	
**DnaJ homolog subfamily A member 1**	*Dnaja1*		1.40	
**Glutaredoxin-1**	*Glrx*		1.42	
**Heat shock protein HSP 90β**	*Hsp90ab1*	2.06	2.45	
**Endoplasmin precursor**	*Hsp90b1*		1.60	
**78 kDa glucose-regulated protein precursor**	*Hspa5*	2.31	1.86	
**Heat-shock cognate 71 kDa protein**	*Hspa8*	2.75	2.76	
**Heat-shock protein β-1**	*Hspb1*		1.76	
**Heat-shock protein β-8**	*Hspb8*		1.93	
**60 kDa heat-shock protein, mitochondrial precursor**	*Hspd1*		2.47	
**Heat-shock protein 105 kDa**	*Hsph1*		2.37	
**ubiquitin C**	*Ubc*	2.86	2.07	4.49
**ubiquitin-conjugating enzyme E2B**	*Ube2b*		1.55	
**protease (prosome, macropain) 26 S subunit, ATPase 1**	*Psmc1*		1.42	
**protease (prosome, macropain) 26 S subunit, ATPase 4**	*Psmc4*		1.50	
**protease (prosome, macropain) 26 S subunit, ATPase 5**	*Psmc5*		1.47	
**proteasome (prosome, macropain) 26 S subunit, non-ATPase, 4**	*Psmd4*		1.44	
**proteasome (prosome, macropain) 26 S subunit, non-ATPase, 11**	*Psmd11*		1.44	
**proteasome (prosome, macropain) 26 S subunit, non-ATPase, 13**	*Psmd13*		2.08	

## Discussion

The major architectural feature of fasting liver is a pronounced decline in cell size (down to ~75% of its fed diameter after 3 days of fasting) rather than a loss of cell number. In addition, the liver's metabolic zonation in upstream, periportal and downstream, pericentral regions remains intact. These findings indicate that the liver can quickly resume its homeostatic functions once feeding resumes. Our microarray data show that the adaptive response of the liver to fasting at the level of gene expression is most pronounced during the early phase, with the upregulation of ammonia detoxification persisting up to 72 hours of fasting. Since the technically similar study of Bauer et al. [22] reported enhanced expression of lipid-catabolizing and urea-cycle enzymes after 24 and 48 h of fasting, the collective data show that the response to fasting in the liver starts already at 12 hours of fasting and becomes maximal between 24 and 48 hours. The hepatic response to total food deprivation, therefore, does not proceed through the global "sugar-fat-protein" sequence that is described for the adaptation on the whole-body level [1-5].

The expression of genes involved in lipid metabolism and ketone-body synthesis, many under PPARα coordination [29,26], was strongly regulated towards fatty-acid oxidation and ketone-body formation. Interestingly, this adaptive response was seen between 12 and 48 hours ([22] and present study) of fasting only, and then faded out. In the rat, this response was recently reported to occur between 3 and 5 days of fasting [30]. Fatty-acid oxidation was accommodated by the identical time frame of the upregulation of the expression of TCA-cycle enzymes and the proteins of the electron-transport chain in response to fasting. The associated oxidative stress and mitochondrial radical formation was apparently sufficiently strong to induce the unfolded-protein (ER stress) response in liver. Thus far, to our knowledge, activation of the unfolded protein response has not been associated with fatty-acid oxidation in the fasting liver, but it is induced by a high-fat diet [31]. Similarly, mitochondria in fasting muscle protect themselves against the oxidative stress that results from fat oxidation [32] by accumulating the uncoupling proteins UCP2 and UCP3 [33,34].

The role of the liver in gluconeogenesis during fasting is well documented [35-37]. However, the expression of enzymes associated with gluconeogenesis was upregulated only during the first day of fasting and was mainly confined to the malate-aspartate shuttle and *Pepck1*. In fact, apart from *Pepck1*, the expression of none of the committed steps in gluconeogenesis was regulated. It is likely that the enhanced expression of TCA-cycle and malate-aspartate shuttle enzymes, and the enhanced expression of *Pepck1 *enhance the flux towards either glucose-6-phosphate or lactate. It is, therefore, remarkable that the expression of glucose-6-phosphatase, a periportal enzyme, and pyruvate kinase, a mainly pericentral enzyme, are not regulated, while the expression of lactate dehydrogenase is only upregulated at 12 and 24 hours. Similarly, the expression and activity of glucose-6-phosphatase in rat liver are upregulated mildly during the first 48 hours of fasting only [38]. Our unpublished data show a similar response in the kidneys of fasting mice, in which *Pepck1 *is 2–3 fold upregulated at all time points, whereas glucose-6-phosphatase is not regulated. The pronounced accumulation of glycogen in pericentral hepatocytes starting after 24 hours of fasting, which was also observed in 72- and 96-hour fasted rats [39], indicates that pericentral hepatocytes, which do not express glucose-6-phosphatase [40,41], channel glucose-6-phosphate towards glycogen. Since all relevant enzymes are also expressed in periportal hepatocytes, which do not accumulate glycogen, we assume that these hepatocytes contain enough glucose-6-phosphatase to produce glucose.

The liver produces ~60% of the newly produced glucose in starvation, while the kidneys account for ~40% [13]. A recent, but controversial series of experiments suggest that, in addition to the liver [35-37] and kidney [35,42], the small intestine also has the capacity to produce glucose upon prolonged fasting [43,6]. It contributes indirectly, by providing lactate and alanine to the liver in short-term fasting [17,44], and directly by the production of glucose [6] (perhaps up to 27% of whole-body glucose production in extended fasting in the rat [18]). The concept is controversial, since other studies were unable to detect glucose formation from glutamine in the isolated small intestine of 72 hours fasted rats [45]. Furthermore, the expression of the key gluconeogenic enzyme phosphoenolpyruvate kinase (*Pepck1*) in the mouse small intestine was reported to amount to only 0.05–1% of that in the liver after 12 h hours of fasting [46], also arguing against intestinal gluconeogenesis. We, therefore, compared *Pepck *mRNA levels in these two organs by qPCR in the fed and 3-days fasting condition (Table 2). While *Pepck1 *expression in the gut at 12 hours of fasting only amounted to ~5% of that in liver, its expression increased to 18 and 53% of that in the liver after 24 and 72 hours of fasting, respectively. This finding demonstrates that the issue of intestinal gluconeogenesis during prolonged fasting deserves additional study.

The plasma concentrations of both glucose and lactate remained unchanged during the first 24 hours of fasting, declined temporarily by 35–40% at 48 hours, and returned to control values between 48 and 72 hours. The maintenance of normal concentrations of glucose and lactate during the first 24 hours of fasting is most likely the result of gluconeogenesis in the liver and kidney. Since the expression of enzymes that are shared by the glycolytic and gluconeogenic pathways, declines after 24 hours of fasting, the observed decline at 48 hours may represent a declining contribution of the liver to gluconeogenesis. As we argued earlier, the accumulation of glycogen in the pericentral hepatocytes between 24 and 72 hours of fasting indicates that gluconeogenic intermediates flow towards glucose-6-phosphate and accumulate as glycogen due to the low expression of glucose-6-phosphatase in these hepatocytes. Most likely, a similar or higher flow of gluconeogenic intermediates is present in the periportal hepatocytes, but is exported as glucose due to the high concentration of glucose-6-phosphatese in these cells. Furthermore, the putative production of glucose in the 72-hour fasted intestine can also contribute to the circulating glucose level.

The urea-cycle enzymes distinguish themselves from most other genes in the liver in that they were upregulated in expression throughout the period of fasting that was studied. Furthermore, cytosolic glutamate-oxaloacetate transaminase, which mediates the availability of aspartate to the urea-cycle enzyme argininosuccinate synthetase, was also strongly upregulated at 72 hours. Similarly, *Oat *and *Prodh*, which supply glutamate to glutamine synthetase for glutamine synthesis in pericentral hepatocytes, were strongly upregulated at all time points studied, but glutamine synthetase itself was not regulated (and even downregulated in another study [22]). Since few amino-acid catabolizing enzymes were upregulated (the exception being the metabolism of sulphur-containing amino acids), most amino-groups were probably carried to the liver as alanine or glutamine, although neither glutamate-pyruvate transaminase nor liver glutaminase was upregulated. The coordinate control of ammonia detoxification and the source of ammonia during prolonged fasting therefore deserve attention.

An important question is to what extent we can extrapolate the observations in a small mammal like the mouse to larger animals like humans. The ability to tolerate the absence of food does indeed decline with body size: in the mouse the maximum duration of fasting is 4 days [47], in rat 12–15 days [48], in children 4 weeks and in adult humans 8–9 weeks [49,13]. Qualitatively, however, the response to fasting is probably comparable between these mammals, as long as the time scale is adjusted to the size of the animal. Rather than questioning the comparability of small and large animals, our data question whether the implicit extrapolation of the "sugars-fats-proteins" succession of energy substrates during fasting that is based on whole-body energy expenditure [1,50] to individual organs is valid. Microarray studies in rodents that have prospected the adaptive response to fasting of the small intestine [6], liver ([22] and present study), muscle [23,24,51], and a more limited study in kidney focusing on circadian differences in gene expression [52], reveal a different scenario. Muscle and kidney respond to fasting with a progressive change over time in mRNA concentrations of enzymes involved in protein, carbohydrate and fat metabolism. The response in liver peaked at 24–48 hours of fasting in mouse, while most adaptive changes had abated by 72 hours. The intestine, finally, showed an early, but temporary peak of adaptive changes in amino-acid, carbohydrate and fat metabolism at 12 hours of fasting, while a late response, existing almost exclusively of amino-acid catabolizing and gluconeogenic enzymes, gradually developed towards 72 hours of fasting. These differences in pattern and amplitude of gene expression change in different organs can be used to look for circulating biomarkers that reflect the functions of organs during adaptive responses.

## Conclusion

Based on whole-body energy expenditure, the "sugars-fats-proteins" sequence of energy substrates during fasting was suggested. In our extensive microarray studies of the response to fasting in the gut [6] and liver (present study), we found no support for this intuitively attractive model at the individual organ level. The liver markedly differed from the biphasic response pattern in the small intestine (with peaks at 12 and 72 hours) in that its adaptive response peaked at 24–48 hours of fasting, while most adaptive changes had abated by 72 hours. Expression profiling and pathway analysis revealed that genes involved in amino-acid, lipid, carbohydrate and energy metabolism responded most significantly to fasting, with no temporal separation between them. Even though the liver lost 50% of its initial weight during 3 days of fasting, its basic morphology remained preserved, showing that the liver can quickly resume its homeostatic functions when feeding resumes.

## Methods

### Animals and tissues

Livers were harvested from the same mice that were used to study the effects of fasting on the small intestine [6]. Briefly, 6 week-old male FVB mice (Charles River, Maastricht, The Netherlands) were fasted for 0, 12, 24, or 72 hours before sacrifice (N ≥ 8 per group). The animals were kept in metabolic cages to prevent the consumption of bedding and were kept warm with an infrared lamp. Body weight was determined daily. The daily rate of body or organ mass loss was calculated as described [53]. The animals were sacrificed between 9:00 and 10:00 a.m. by cervical dislocation. The liver was isolated quickly, freed from the gall bladder, cut into pieces and either snap-frozen in liquid N_2 _and stored at -80°C, or fixed overnight at 4°C in 4% buffered formaldehyde or a mixture of methanol, acetone, and water (2:2:1 by volume). The study followed the Dutch guidelines for the use of experimental animals and was approved by the AMC Animal Experiments Committee.

### RNA isolation and quantification

Total liver RNA was extracted from frozen tissue with TRIzol reagent (Invitrogen, Breda, The Netherlands). The RNA quality was assessed using the RNA 6000 Nano LabChip^® ^Kit in an Agilent 2100 bioanalyzer (Agilent Technologies, Palo Alto, USA). Additional mRNA quantification for *Cps *(not present on the microarray) and the qPCR validation of the changes derived from the microarray read-outs was performed as described [54] (Supplementary Table 2, Additional file 1). The gene-specific primer sequences are shown in Supplementary Table 3 (Additional file 1). mRNA concentration was calculated using the LinReg program [55]. The significance of the qPCR data was assessed by Student's t-test.

### Microarrays

Three microarrays (Mouse Development Oligo Microarrays G4120A; 22 K; Agilent) per experimental condition and a robust reference design were used [56]. Per microarray, 20 μg mRNA, pooled from 2 livers, was reverse transcribed with Cy3-labelled dCTP (Perkin Elmer, Boston, USA), using the Agilent Fluorescent Direct Label Kit. Cy5-labeled cDNA, produced from RNA pooled from the livers of 6 fed animals, served as the common reference across all arrays. Hybridized cDNAs were detected with Agilent's dual-laser microarray-slide scanner and the data retrieved with Agilent's Feature Extraction software 6.1.1.  The data discussed in this publication have been deposited in NCBIs Gene Expression Omnibus (GEO; [66]) and are accessible through GEO Series accession number GSE10653.

### Data analysis

The data were processed and analyzed as described [6]. In brief, background-subtracted intensities were calculated using foreground and background median signals, and normalized with the quantile normalization method. An ANOVA model was applied to the common reference channel only, to remove outliers and local artefacts, and detect non-uniform hybridization [57]. Differentially expressed genes were identified with the Split-Factor ANOVA directly by comparing the green (experimental) and red (reference) signals, and indirectly, across-arrays, by comparing the Cy3 signals of starved and fed animals. A consensus between the direct and across-array ANOVA ensures that the final results do not suffer from either dye-gene effects or array-specific noise. Genes that received a concordant significance call in 2 out of 3 microarrays (P < 0.01) from both the direct and across-array split-factor ANOVA were taken into further consideration. Given the high sensitivity of Agilent arrays [58], we opted for 1.4-fold change as inclusion criterion for a gene.

To perform cluster analysis, Pearson correlation was set as distance measure and complete linkage as agglomeration method. The normalized log-ratio (Cy5/Cy3) expression values of the top 500 most differentially expressed genes between fasted and normal fed mice were used to calculate the correlation between samples. R/Bioconductor [59] was used to create the clusters.

Pathway, network and gene-set enrichment analyses were applied system-wide, using the MetaCore™ suit (GeneGo, Inc., St. Joseph, MI, USA) [60,61]. The significance of changes in expression in pathways or networks is based on the degree of overlap between the user's dataset and a set of genes corresponding to a network or pathway queried. The problem is cast as the probability that a randomly obtained overlap of a certain size between the user's set and a network/pathway follows a hypergeometric distribution. Additionally, pathway analysis and visualisation was performed using GenMAPP [62] (2.0 β-version) software (Gladstone Institutes, UCSF, San Francisco, USA). In all applications, P < 0.01 and ≥ 1.4-fold change were used as inclusion criteria.

To assess the significance of the results other than microarray data, ANOVA and Student's t-test were employed. The error bars in the figures represent the standard error of the mean (SEM).

### Histology and immunohistochemistry

Sections were stained with hematoxylin and azophloxine, or immunohistochemically as described [6,63]. Monoclonal anti-glutamine synthetase (Transduction Laboratories, Lexington, KY) and polyclonal anti-carbamoylphosphate synthetase (CPS, [64]) antibodies were used. Antibody binding was visualized with goat anti-mouse or goat anti-rabbit IgG, both coupled to alkaline phosphatase (Sigma).

Periodic acid-Schiff (PAS) staining was performed to visualize glycogen in the liver. The sections were incubated for 30 minutes in 0.5% periodic acid, followed by incubation in Schiff's reagent for 30 minutes, and counterstained in haematoxylin for 6 minutes. The identity of glycogen was verified by predigesting a serial section with 0.1% α-amylase for 45 minutes prior to staining.

### Biochemical measurements

Blood ammonia levels were determined immediately after collecting blood from the caval vein, using Ammonia Checker II (model AA-4120, Kyoto Daiichi Kagaku Co., Japan) and the corresponding Ammonia test kit II (Arkray, Inc., Japan).

For determination of free amino acids, 10 μL of plasma (blood was collected in heparin-containing tubes, and centrifuged for 5 min at 14,000 rpm at 4°C) was mixed with 0.8 mg of lyophilized sulphosalicylic acid, centrifuged, and the supernatant stored at -80°C. Amino-acid analysis was performed using a gradient reverse-phase HPLC system, with precolumn derivatization with o-phtaladehyde (Pierce) and 3-mercaptopropionic acid (Sigma), and fluorescence detection [65]. Separation was performed using an Omnisphere 3 column (Varian, Middelburg, The Netherlands).

For glucose and lactate measurements, plasma was acidified with 1 volume 2 M (12%) perchloric acid, centrifuged for 15 minutes, and neutralized with 1 M MES/2 M KOH. Plasma glucose and lactate concentrations were measured enzymatically, using the NOVOstar reader (BMG Labtech GmbH, Offenburg, Germany).

## Abbreviations

*Acaa1*: acetyl-coenzyme A acyltransferase 1; *Acad/-v,-l,-m,-sh*: acyl-coenzyme A dehydrogenase/very long, long, medium and short chain; *Acaddm*: acetyl-coenzyme A dehydrogenase, medium chain; *Aco2*: aconitase 2; *Aip*: AH receptor-interacting protein; *Aldoa*: aldolase 1A isoform; ANOVA: analysis of variance; *Asl*, *Arly*: argininosuccinate lyase; *Ass1*, *Assy*: argininosuccinate synthetase 1; *Atf4*: Cyclic AMP-dependent transcription factor ATF-4 *Atp5a1*: ATP synthase subunit α; *Atp5d*: ATP synthase δ chain; *Atp5g1*: ATP synthase lipid-binding protein; *Cac*, *Slc25a20*: carnitine/acylcarnitine fatty-acid translocase; *Calr*: calreticulin precursor; *Cat*: catalase; *Cps*: carbamoylphosphate synthetase; *Cpt2*: carnitine palmitoyltransferase 2; Cy3, Cy5: fluorescent dyes of the cyanine dye family; *Dlst*: dihydrolipoamide S-succinyltransferase; *Dnaja1*: DnaJ homolog subfamily A member 1; *Echdc3*: enoyl coenzyme A hydratase domain containing 3; *Egr1*: early growth response protein 1; *Ehhadh*: enoyl-coenzyme A, hydratase/3-hydroxyacyl-coenzyme; *Eno1*: enolase 1α; ER: endoplasmic reticulum; *Fh1*: fumarate hydratase 1; FVB: mouse strain sensitive to Friend leukaemia virus B; *Gapdh*: glyceraldehyde-3-phosphate dehydrogenase; *Glrx*: glutaredoxin-1; *Got1*: glutamate-oxaloacetate transaminase cytosolic; *Got2*: glutamate-oxaloacetate transaminase mitochondrial; *Gpi1*: glucosephosphate isomerase 1; GS, *Glns*, *Glul*: glutamine synthetase; HA: hematoxylin, azophloxin; *Hadhb*-trifunctional protein, β-subunit; *Hadhsc*: hydroxyacyl-coenzyme A dehydrogenase, short chain; *Hmgcl*: 3-hydroxy-3-methylglutaryl-coenzyme A lyase; *Hmgcs2*: 3-hydroxy-3-methylglutaryl-Coenzyme A synthase 2; *Hsp90ab1*: heat shock protein HSP 90-beta; *Hsp90b1*: endoplasmin precursor; *Hspa5*: 78 kDa glucose-regulated protein precursor; *Hspa8*: heat shock cognate 71 kDa protein; *Hspb1*: heat-shock protein beta-1; *Hspb8*: heat-shock protein beta-8; *Hspdv*: 160 kDa heat shock protein, mitochondrial precursor; *Hsph1*: heat-shock protein 105 kDa; *Idh3b*: isocitrate dehydrogenase 3β (NAD+); KOH: potassium hydroxide; *Mapk14*: mitogen-activated protein kinase 14; *Mdh1*: malate dehydrogenase 1; MES: 2-(N-Morpholino)ethanesulfonic acid; *Ndufa10*: NADH dehydrogenase [ubiquinone] 1α subcomplex subunit 10; *Ndufv*: NADH dehydrogenase [ubiquinone] flavoprotein; *Oat*: ornithine aminotransferase; *Ogdh*: oxoglutarate dehydrogenase; PAS: Periodic acid-Schiff; *Pepck*, *Pck1*: phosphoenolpyruvate carboxykinase 1; *Pgam1*: phosphoglycerate mutase 1; *Pparα*: peroxisome proliferator-activated receptor, *α *isotype; *Prodh*: proline dehydrogenase; *Psmc*: protease (prosome, macropain) 26 S subunit, ATPase; *Psmd*: proteasome (prosome, macropain) 26 S subunit, non-ATPase; qPCR: quantitative polymerase chain reaction; RNA: ribonucleic acid; *Scd1*: stearoyl-Coenzyme A desaturase 1; *Sod1*: superoxide dismutase; TCA: tricarboxylic acid cycle; *Tpi1*: triosephosphate isomerase 1; *UbC*: ubiquitin C; *Ube2b*: ubiquitin-conjugating enzyme E2B; *Ucp2*: uncoupling protein 2; *Uqcrc1*: ubiquinol-cytochrome-c reductase complex core protein 1

## Authors' contributions

MS carried out the biological part of the study and prepared the manuscript. AS contributed to the biological part of the research. DdW performed a part of biochemical measurements. DW designed and carried out a part of the bioinformatics analysis of the data, together with LGP and EVLvT. AvK supervised this part of the study. YN supported the data analysis in the MetaCore suite. TH and WL supervised the biological part of the study.

## Supplementary Material

Additional file 1 **Supplementary tables**. *Supplementary table 1 *shows amino-acid concentrations in plasma after 0, 12, 24, 48 and 72 hours of fasting. *Supplementary table 2 *shows qPCR validation of microarray data. *Supplementary table 3 *contains gene-specific primer sequences, product lengths and annealing temperatures. Click here for file

Additional file 2 **Fold changes in liver response to fasting**. The file contains lists of all the genes significantly regulated in the liver (≥ 1.4 fold) per time point of fasting. Click here for file

Additional file 3 **MetaCore legend**. The file contains a legend for the pathways and the network created in MetaCore suite shown in the Figures 7, 8 and 10. Click here for file
