# Supplementary material for: The transcriptomic signature of fasting murine liver
Source: BMC Genomics. 2008 Nov 6;9:528. doi: 10.1186/1471-2164-9-528 (PMC2588605; doi:10.1186/1471-2164-9-528)
Supplement: Additional file 1 — Supplementary tables. Supplementary table 1 shows amino-acid concentrations in plasma after 0, 12, 24, 48 and 72 hours of fasting. Supplementary table 2 shows qPCR validation of microarray data. Supplementary table 3 contains gene-specific primer sequences, product lengths and annealing temperatures. [file 1471-2164-9-528-S1.doc]

Supplementary Table 1: Amino-acid concentrations in fasting plasma

| **amino**  **acid** | **0h**  ***(µM)*** | **12h**  ***(µM)*** | **0 vs. 12**  ***P-value*** | **24h**  ***(µM)*** | **0 vs. 24**  ***P-value*** | **48h**  ***(µM)*** | **0 vs. 48**  ***P-value*** | **72h**  ***(µM)*** | **0 vs. 72**  ***P-value*** |
| --- | --- | --- | --- | --- | --- | --- | --- | --- | --- |
| **Asn** | 35 | 46 | 8.69E-02 | 43 | **6.75E-05** | 56 | **1.05E-02** | 60 | **1.76E-03** |
| **Ser** | 135 | 77 | **8.88E-04** | 72 | **7.19E-06** | 78 | **9.82E-03** | 166 | 3.43E-01 |
| **Gln** | 553 | 528 | 4.48E-01 | 598 | 1.14E-01 | 589 | 4.46E-01 | 656 | 7.29E-02 |
| **His** | 81 | 63 | **6.22E-03** | 74 | **2.64E-02** | 80 | 9.70E-01 | 83 | 8.16E-01 |
| **Gly** | 87 | 161 | 2.48E-01 | 58 | 1.95E-01 | 43 | **4.66E-02** | 115 | 3.59E-01 |
| **Thr** | 117 | 108 | 5.91E-01 | 114 | 6.01E-01 | 141 | 2.00E-01 | 146 | **4.58E-03** |
| **Cit** | 91 | 55 | 7.07E-02 | 61 | **4.81E-07** | 79 | 2.50E-01 | 74 | **2.38E-02** |
| **Arg** | 99 | 85 | **1.55E-02** | 83 | 5.92E-02 | 115 | 2.62E-01 | 121 | 1.39E-01 |
| **Ala** | 314 | 255 | 6.39E-02 | 232 | **5.66E-03** | 285 | 4.75E-01 | 295 | 5.33E-01 |
| **Tau** | 289 | 334 | 1.61E-01 | 445 | **3.17E-04** | 413 | **2.13E-03** | 494 | **1.45E-05** |
| **Tyr** | 76 | 68 | 6.73E-02 | 69 | **3.37E-02** | 77 | 9.15E-01 | 77 | 7.38E-01 |
| **Val** | 196 | 189 | 7.60E-01 | 232 | **2.07E-02** | 235 | 2.80E-01 | 220 | 1.39E-01 |
| **Met** | 40 | 35 | 6.26E-02 | 41 | 8.36E-01 | 41 | 9.09E-01 | 40 | 9.34E-01 |
| **Ile** | 76 | 90 | 3.93E-01 | 103 | **2.69E-03** | 105 | 6.07E-02 | 93 | 7.44E-02 |
| **Phe** | 78 | 44 | 1.19E-01 | 98 | **9.43E-04** | 101 | 2.90E-01 | 114 | **1.72E-04** |

Amino-acid concentrations in plasma after 0, 12, 24, 48 and 72 hours of fasting are expressed in µM. The significance of the change between the groups is shown by P-values, with the significant changes (P < 0.05) printed in bold.

**Supplementary Table 2: qPCR validation of changes in gene expression detected by microarrays.** Significance threshold is set to P < 0.01 (labeled by asterisks; n = 6).

| **symbol** | **gene name** | **qPCR** | | | | | | **microarray** | | | | | |
| --- | --- | --- | --- | --- | --- | --- | --- | --- | --- | --- | --- | --- | --- |
|  |  | **12h** | ***24h*** | | **72h** | | **12h** | | **24h** | | **72h** | |  |
| ***ApoA4*** | apolipoprotein A4 | 9.2* | | 14.4* | | 21.0* | | 10.4* | | 11.1* | | 19.7* | |
| ***Casp6*** | caspase 6 | 1.3 | | 1.9 | | 0.7 | | 1.3 | | 1.4 | | 1.05 | |
| ***Gs*** | glutamine synthetase | 1.3 | | 1.6 | | 1.3 | | 1.0 | | 1.1 | | 1.1 | |
| ***Nr1h3*** | nuclear receptor subfamily 1 H3 (*Lxr*) | 1.2 | | 1.7* | | 1.3 | | 1.3 | | 1.4* | | 1.1 | |
| ***Pepck1*** | phosphoenolpyruvate carboxykinase 1 | 3.3 | | 3.2* | | 2.9* | | 2.1* | | 2.5* | | 2.7* | |
| ***Rxrα*** | retinoid x receptor α | 2.1 | | 5.1 | | 3.5 | | 1.2 | | 1.3 | | 1.1 | |

**Supplementary Table 3:** **Gene-specific primer sequences, product lengths and annealing temperatures.**

| **gene** | **primer sequence (5’→ 3’)** | **product length (bp)** | **temp(˚C)** |
| --- | --- | --- | --- |
| ***18S*** | F: TTCGGAACTGAGGCCATGAT  R: CGAACCTCCGACTTTCGTTCT | 132 | 58 |
| ***Pck1*** | F: GCCAAGCTCACGCCCATC  R: CTCACGATTGTGCCGCTAT | 329 | 56 |
| ***Cps*** | F: TGGGCCATCTCAGGAAGGAC  R: CTGCCTGTAGTGGAACAGAC | 253 | 58 |
| ***Gs*** | F: CCACCTCAGCAAGTTCCC  R: GGCTTCCGGTTATACTTG | 318 | 55 |
| ***Casp6*** | F: GTGTTCGATCCAGCCGAG  R: GGCGTCGTATGCGTAAAC | 309 | 55 |
| ***Rxrα*** | F: GCACGTACACCGGAACA  R: CGCTTCTAGTGACGCATA | 217 | 53 |
| ***ApoA4*** | F: GGAGGCTGTAGAACAGTTTCAGAAG  R: TCCCCAAGTTTGTCCTGGAA | 94 | 60 |
| ***Nr1h3*** | F: TCAGCATCTTCTCTGCAGACCGG  R: TCATTAGCATCCGTGGGAACA | 144 | 60 |
