# Supplementary material for: The transcriptomic signature of fasting murine liver
Source: BMC Genomics. 2008 Nov 6;9:528. doi: 10.1186/1471-2164-9-528 (PMC2588605; doi:10.1186/1471-2164-9-528)
Supplement: Additional file 3 — MetaCore legend. The file contains a legend for the pathways and the network created in MetaCore suite shown in the Figures 7, 8 and 10. [file 1471-2164-9-528-S3.pdf]

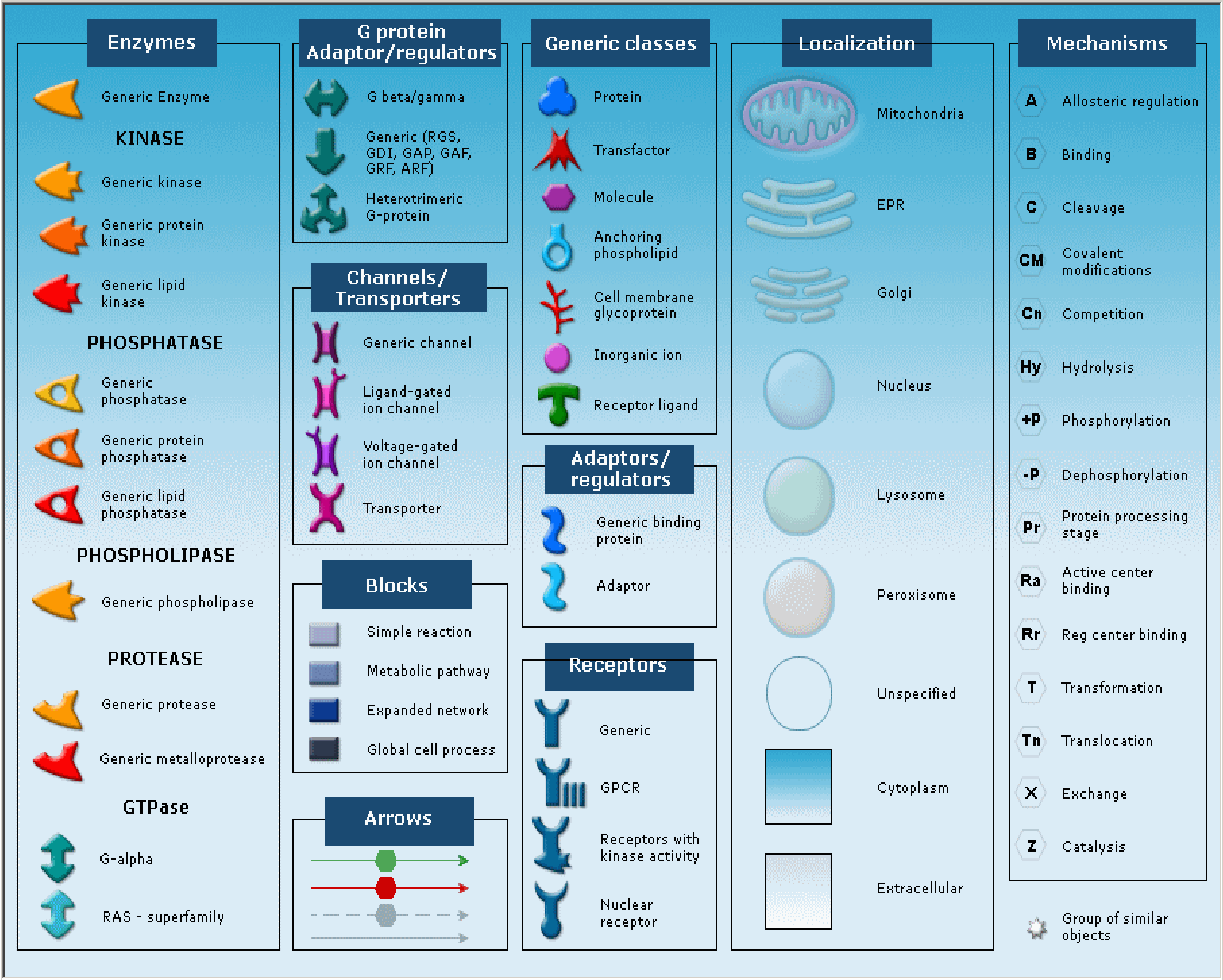

Enzymes
Generic Enzyme
KINASE
Generic kinase
Generic protein kinase
Generic lipid kinase
PHOSPHATASE
Generic phosphatase
Generic protein phosphatase
Generic lipid phosphatase
PHOSPHOLIPASE
Generic phospholipase
PROTEASE
Generic protease
Generic metalloprotease
GTPase
G-alpha
RAS - superfamily
G protein Adaptor/regulators
G beta/gamma
Generic (RGS, GDI, GAP, GAF, GRF, ARF)
Heterotrimeric G-protein
Channels/ Transporters
Generic channel
Ligand-gated ion channel
Voltage-gated ion channel
Transporter
Blocks
Simple reaction
Metabolic pathway
Expanded network
Global cell process
Arrows
Generic classes
Protein
Transfactor
Molecule
Anchoring phospholipid
Cell membrane glycoprotein
Inorganic ion
Receptor ligand
Adaptors/ regulators
Generic binding protein
Adaptor
Receptors
Generic
GPCR
Receptors with kinase activity
Nuclear receptor
Localization
Mitochondria
EPR
Golgi
Nucleus
Lysosome
Peroxisome
Unspecified
Cytoplasm
Extracellular
Mechanisms
A Allosteric regulation
B Binding
C Cleavage
CM Covalent modifications
Cn Competition
Hy Hydrolysis
+P Phosphorylation
-P Dephosphorylation
Pr Protein processing stage
Ra Active center binding
Rr Reg center binding
T Transformation
Tn Translocation
X Exchange
Z Catalysis
Group of similar objects
